# AI-assisted diffuse correlation tomography for identifying breast cancer

**DOI:** 10.1117/1.JBO.30.5.055001

**Published:** 2025-05-16

**Authors:** Ruizhi Zhang, Jianju Lu, Wenqi Di, Zhiguo Gui, Shun Wan Chan, Fengbao Yang, Yu Shang

**Affiliations:** a North University of China, State Key Laboratory of Dynamic Measurement Technology, Taiyuan, China; b Affiliated Hospital of Jiaxing University, The First Hospital of Jiaxing, Jiaxing, China; c Technological and Higher Education Institute of Hong Kong, Department of Food and Health Sciences, Hong Kong, China; d Dongguan University of Technology, School of Life and Health Technology, Dongguan, China

**Keywords:** artificial intelligence, breast cancer, clinical imaging, microvascular blood flow, diagnosis

## Abstract

**Significance:**

Diffuse correlation tomography (DCT) is an emerging technique for the noninvasive measurement of breast microvascular blood flow, whereas its capability to categorize benign and malignant breast lesions has not been extensively validated thus far, due to the difficulties in instrumentation, image reconstruction algorithms, and appropriate approaches for imaging analyses.

**Aim:**

This artificial intelligence (AI)-assisted DCT instrumentation was constructed based on a unique source–detector array and image reconstruction algorithm.

**Approach:**

The DCT images of breasts were obtained from 61 females, and AI models were utilized to classify breast lesions. During this process, the blood flow images were either extracted as feature parameters or as global inputs to the AI models.

**Results:**

As the validations of DCT instrumentation, the blood flow images obtained from longitudinal monitoring of healthy subjects demonstrated the stability of DCT measurements. For patients with breast diseases, comprehensive analyses yield an AI-assisted classification with excellent performance for distinguishing between benign and malignant breast lesions, at an accuracy of 97%.

**Conclusions:**

The AI-assisted DCT reflects functional abnormalities that are associated with cancellous-induced high metabolic demands, thus demonstrating the great potential for early diagnosis and timely therapeutic assessment of breast cancer, e.g., prior to the tumor formation or proliferation of microvascular networks.

## 1 Introduction

For adult women, breast cancer is the most frequent occurrence of malignant tumors and the leading cause of cancerous death worldwide.^1^ The breast cancer mortality rates in many high-income countries at historically high incidence rates have shown a decline since the early 1990s; meanwhile, the developing countries have seen a rapid increase in both breast cancer incidence and mortality, and many sub-Saharan African countries rank the highest worldwide in terms of breast cancer mortality.^2^ The survival outcomes of breast cancer exhibit substantial geographic variability, primarily due to the difference in healthcare services and disease screening. Early detection, precise therapy, and comprehensive medical management are the three key factors in reducing breast cancer mortality.^3^

The early detection of breast cancer can be achieved through various approaches to breast screening.^4^ The mammography screening modality, recommended by the WHO for women aged 50–69 years at average risk for breast cancer, is highly effective but limited to well-resourced clinical settings.^1^ In addition, the mammography exhibits insufficient sensitivity when being applied to dense breasts.^5^ Another imaging technique for breast cancer diagnosis, ultrasound imaging, is limited by low specificity.^6^ Magnetic resonance imaging (MRI) has a relatively long examination time and requires heavy and expensive instruments.^7^ Positron emission tomography has radioactive risk and is at an extremely high cost.^8^ The gold standard for the ultimate identification of breast cancer is biopsy, which is invasive and not favorable to early detection.^9^ Despite the advanced developments in imaging techniques, the issue of low positive predictive value for biopsy recommendation persists.^10^

The aforementioned imaging techniques primarily focus on morphological changes of breast tumors, which typically occur posterior to the microvascular hemodynamic alterations resulting from the enhanced metabolic demand that is associated with autonomic tumor growth.^11^^,^^12^ On the other hand, functional optical imaging techniques that capture microvascular hemodynamics (such as blood flow perfusion, oxygen, and oxygen metabolism) hold the potential to facilitate earlier detection of breast tumors.^13^^–^^15^ Generally, breast optical images require the injection of photons from point-like sources through optical fibers, with the escaped photons being detected at one or multiple appropriate detector locations. The instruments for optical breast imaging and spectroscopy can be divided into four primary categories according to their measurement geometry. In the first approach, the breast is compressed between two parallel plates, resembling the geometry similar to that employed in X-ray mammography.^16^ However, this approach has several drawbacks such as patient discomfort caused by tissue compression as well as the limited suitability for patients with small breasts. The second approach involves imaging the freely-pending breast in a prone position, resembling the geometric configuration similar to that utilized in breast MRI.^17^ This circular-based configuration minimizes patient discomfort but is constrained by the bulky and non-portable instrumentation, precluding it from routine clinical setting and diagnosis. The third approach involves the utilization of a noncontact probe spatially scanning over the breast area, which is suitable for breast tissue afflicted with ulcers and infectious complications.^18^ However, this kind of noncontact measurement requires the patient to maintain a stationary position for a long period of sequential scanning (∼20  min), thereby inevitably introducing motion artifacts to the image reconstruction. The fourth approach is the use of a handheld probe that can be strategically positioned at specific locations on the breast or moved over its surface, similar to a breast ultrasound transducer.^19^ This sub-surface imaging configuration enhances patient comfortability and portability, whereas the handheld measurement is influenced by the operator’s experience.

The functional measurement/imaging modality of near-infrared (NIR) diffuse optical spectroscopy/tomography (DOS/DOT) has demonstrated remarkable advantages in breast cancer diagnosis and therapeutical monitoring.^20^^–^^22^ DOS/DOT employs the NIR light diffused within the biological tissue to obtain blood oxygenation information, including oxy- and deoxy-hemoglobin, total hemoglobin, and oxygen saturation. DOS/DOT has been extensively adopted by integrating the aforementioned detection methods as well as artificial intelligence (AI) models to discriminate breast images of malignant and benign lesions. A few groups have utilized machine learning (ML) models, such as regression tree algorithm,^23^ logistic regression model,^24^^,^^25^ and support vector machine (SVM) model,^26^ for accurate classification of DOS imaging (DOSI) and DOT images, resulting in favorable diagnostic performance. One group adopted deep learning (DL) networks, such as convolutional neural networks (CNNs), to classify DOT images, obtaining a classification accuracy of 90.2%.^27^ Furthermore, several groups employed a fusion strategy with multiple models to classify DOT images and ultrasound images of lesions, obtaining higher diagnostic performance.^28^^–^^30^ Although DOS/DOT images are promising for breast cancer diagnosis, tissue oxygenation measured/imaged by DOS/DOT solely reflects the static equilibrium between oxygen supply and consumption and does not directly reflect tumor-induced angiogenesis or alterations over the microcirculatory network.

A non-ionizing and noninvasive functional measurement/imaging modality, diffuse correlation spectroscopy/tomography (DCS/DCT) has been emerging in recent years for assessing the microvasculature blood flow in various healthy and diseased human tissues,^13^^,^^31^^,^^32^ including breast cancer.^33^ DCS/DCT employs a long-coherence NIR light to quantify microvascular blood flow index (BFI), which is directly relevant to tumor-induced changes caused by moving scatterers (primarily red blood cells) in a microcirculatory network. The BFI contrasts measured by DCS have shown significant differences among malignant lesions, benign lesions, and surrounding breast tissue.^34^^–^^36^ Compared with the DCS, which only allows for blood flow measurements in a few individual locations on the breast, DCT offers abundant tomographic blood flow images. Nevertheless, few studies have reported DCT clinical breast applications in a small cohort of patients with breast cancer.^37^^,^^38^ The capability of DCT for differentiating malignant and benign breast lesions in a large number of women has not been extensively investigated, partly due to the difficulties in the constructions of a specific DCT system (i.e., a smart integration of hardware components, data acquisition configuration, source–detector (S-D) array on the probe as well as image reconstruction algorithm) that could fast and robustly collect and process the photon signals from the human breast. On the other hand, computer-aided diagnosis with AI models has been used as a pivotal tool for automatically identifying malignant tumors in conventional imaging modalities (mammography, ultrasound, MRI),^39^^,^^40^ supporting the feasibility of combining AI models with DCT data to enhance the accuracy, efficiency, and reproducibility for diagnosis of breast lesions.

In this study, a DCT system was constructed, along with a custom-made optical probe and an 8×8 channel optical switch, specifically for collecting the photon signals from the human breast. Owing to these instrumentation innovations, we successfully collected the DCT images from a relatively large group of 60 women with breast lesions (a woman with three benign lesions and 59 women, 38 with benign lesions, and 21 with malignant lesions). Furthermore, seven different AI models, including the ML model, CNN model, transfer learning (TL) model, and fusion models, were established to classify DCT images into malignant or benign cases. These models were compared with evaluation metrics to determine the performance of different AI approaches for breast cancer diagnosis, based on the DCT image dataset containing a total of 59 subjects.

## 2 Materials and Methods

### 2.1 DCT Hardware System

The hardware system and procedures of DCT data acquisition are depicted in Supplementary Material and elsewhere.^41^^,^^42^ Briefly, the laser-emitted NIR light into the breast tissue via source fibers. After being diffused within the tissue, a portion of the escaped photons were collected in parallel by six single-photon detectors via single-mode detector fibers. The correlator read the outputs of six single-photon detectors and calculated six normalized light intensity temporal autocorrelation curves $\left[g_{2}(\tau )\right]$, which were subsequently transformed into the normalized light electric field autocorrelation functions $\left[g_{1}(\tau )\right]$ via Siegert relation in the data processing module. The $g_{1}(\tau )$ function was measured by each S-D fiber pair and recorded at a 1-Hz rate for 15 s. After the optical switch completed eight switching operations, the total amount of data acquisition was 720 (i.e., 6×8×15) autocorrelation curves within the 2-min acquisition time (i.e., 8×15  s). To minimize the influence of physiological noise (such as breathing and slight movement) and ambient noise on optical measurements, the BFI values obtained from each S-D pair within a 15-s interval were averaged as the sampling data to reconstruct the DCT image.

### 2.2 Reconstruction and Processing of DCT Images

The three-dimensional tomographic DCT images of BFI were reconstructed using the Nth-order linear DCT (NL-DCT) algorithm that was previously established by our laboratory.^42^ The NL-DCT combines the integral form of $g_{1}(\tau )$ with the Nth-order Taylor polynomials, meanwhile accounting for tissue heterogeneity and irregular geometry through comprehensive consideration of photon trajectory information. This approach differs from the conventional analytical solution and finite element method aiming to seek solutions for correlation diffusion equation. First, we established a three-dimensional voxel model to Monte Carlo simulate^43^ diffuse light propagation in the breast tissue (at the size of $8\times 8\times 3\;{\mathrm{cm}}^{3}$, wherein each voxel covers an area of $0.5\times 0.5\times 0.5\;{\mathrm{cm}}^{3}$), aiming to acquire photon transmission information for image reconstruction. During the light Monte Carlo simulations, optical properties were set to be consistent with those of the human breast (i.e., the reduced scattering coefficient equal to $8.0\;{\mathrm{cm}}^{-1}$, absorption coefficient equal to $0.05\;{\mathrm{cm}}^{-1}$, refractive index equal to 1.37, and anisotropy factors equal to 0.9). By assuming that we have M sources (m=1,…,M) to launch photons into tissue and have J detectors (j=1,…,J) to collect the photons that escaped from the tissue, the $g_{1}(\tau )$ from (mth, jth) S-D fiber pairs at multiple delay time τ can be expressed in discrete forms of first-order [N=1, shown in Eq. (1)] and Nth-order [N>1, shown in Eq. (2)] approximations, as follows:

$$g_{1}(m,j,\tau )-1=\tau \overset{n}{\underset{i=1}{\sum }}\alpha D_{B}(i)\overset{Q}{\underset{q=1}{\sum }}-2w(q,m,j)k_{0}^{2}(i)s(i,q,m,j)\mu_{s}^{\prime }(i).$$ (1)

$$g_{1}(m,j,\tau )-1-\overset{N}{\underset{k=2}{\sum }}\frac{\overset{Q}{\underset{q=1}{\sum }}w(q,m,j)(-2\overset{n}{\underset{i=1}{\sum }}k_{0}^{2}(i)\alpha D_{B}(i)s(i,q,m,j)\mu_{s}^{\prime }(i){)}^{k}}{k!}\tau^{k}\;=\tau \overset{n}{\underset{i=1}{\sum }}\alpha D_{B}(i)\overset{Q}{\underset{q=1}{\sum }}-2w(q,m,j)k_{0}^{2}(i)s(i,q,m,j)\mu_{s}^{\prime }(i).$$ (2)

Here, the variable $\alpha D_{B}(i)$ is the BFI of the ith voxel of the tissue model with a total of n voxels. The variable w(q,m,j) is the photon weight of the qth (q=1,…,Q) photon packet from (mth, jth) S-D pair. The variable $k_{0}(i)$ is the light wave vector amplitude of ith voxel. The variable s(i,q,m,j) is the path length of the qth photon packet from (mth, jth) S-D pair within the ith voxel. The variable $\mu_{s}^{\prime }(i)$ is the reduced scattering coefficient of the ith voxel. The variable k is the order of Taylor polynomials. Both w(q,m,j) and s(i,q,m,j) can be estimated from light Monte Carlo simulations in the tissue.

We further adopted the Bregman-TV algorithm [Eq. (3)] to reduce the ill condition during the process of blood flow image reconstruction. Bregman algorithm offers a solution for non-differentiable equations, whereas the total variation (TV) as a regularization term effectively promotes uniformity in non-edge regions without compromising edge preservation.

$$\alpha D_{B}^{*}=\arg\;\min{\left\|\alpha D_{B}\right\|}_{TV}\;+\frac{\mu }{2}\|\alpha D_{B}\overset{Q}{\underset{q=1}{\sum }}-2w(q,m,j)k_{0}^{2}(i)s(i,q,m,j)\mu_{s}^{\prime }(i)-b{\|}_{2}^{2}\;\mathrm{s.t.}\;\alpha D_{B}\geq 0.$$ (3)

Here, $\alpha D_{B}^{*}$ is BFI constrained by Bregman-TV. The variable b is the slope extracted from $g_{1}(\tau )$.

To enhance the classification efficiency by reducing the inter-subject variability and the intra-subject heterogeneity, we performed a normalization scheme [Eq. (4)].

$$\alpha D_{B}(i{)}^{**}=\frac{\alpha D_{B}^{*}(i)}{\frac{1}{n}\overset{n}{\underset{ii=1}{\sum }}\alpha D_{B}^{*}(ii)}.$$ (4)

Here, variable $\alpha D_{B}(i{)}^{**}$ represents the relative BFI (rBFI) at the ith voxel.

The two-dimensional DCT image for classification purposes has an original pixel size of 16×16, located at z=2. Thereafter, the 2D blood flow image was converted to a pixel at a size of 150×150, using an adjacent interpolation approach. The interpolated blood flow image was then imported into an open-source software (ParaView^44^) for improved visualization, by utilizing the colormap type “Cool to Warm”. At this stage, the DCT image, originally in grayscale, was transformed into an RGB image. The Cool to Warm method mapped low and high grayscale values into cool and warm colors, respectively. The output RGB blood flow image was then saved as a TIFF format file. The TIFF-formatted DCT image was resized to a pixel size of 224×224 using the “imresize” function in MATLAB. As this is an RGB image, it comprises three channels—red, green, and blue—resulting in dimensions of 3×224×224.

### 2.3 Participants

This study was approved by the Ethics Committees of both the First Hospital of Jiaxing and the North University of China, adhering to the Declaration of Helsinki. Informed consent forms were obtained from all participants. The maximal tumor sizes were not exceeded and adhered to the tumor size guidelines established by Ethics Committees. A total of 61 female subjects were recruited for this study. One subject (25 years) was healthy without known breast diseases. One subject (26 years) with three benign lesions was diagnosed by MRI and classified as BI-RADS category 2 (i.e., the only benign subject who was measured by both MRI and DCT). The DCT images collected from a total of 59 female subjects (38 with benign lesions and 21 with malignant lesions) were utilized to evaluate the proposed diagnostic classification strategy. The inclusion criteria of 59 subjects were as follows: women aged over 18 years who presented at a symptomatic breast clinic with a palpable lump, and only one suspicious breast lesion was detected through conventional techniques, such as core biopsy (performed longer than 4 weeks prior to DCT measurement to avoid blood flow artifacts from bruising), ultrasound (BI-RADS category 2 to 5), or MRI (lesion in the image identified by two radiologists with more than 10 years of diagnostic experience), not pregnant and without light-sensitive medications. In the benign group (i.e., negative samples, mean age 45 years; range 23 to 73 years; average maximal tumor size of 11.4±8.4  mm), 29% were fibroadenomas, 13% were proliferative lesions, 16% were cysts confirmed by biopsy, and 42% were benign lesions diagnosed by ultrasound with BI-RADS classifying as category 2. In the malignant group (i.e., positive samples, mean age 48 years; range 23 to 65 years; average maximum tumor size of 20.4±9.7  mm), 48% were invasive ductal carcinoma, 4% was invasive lobular carcinoma confirmed by biopsy, and 48% were malignant lesions diagnosed by MRI.

### 2.4 AI Models

**Fig. 1:**
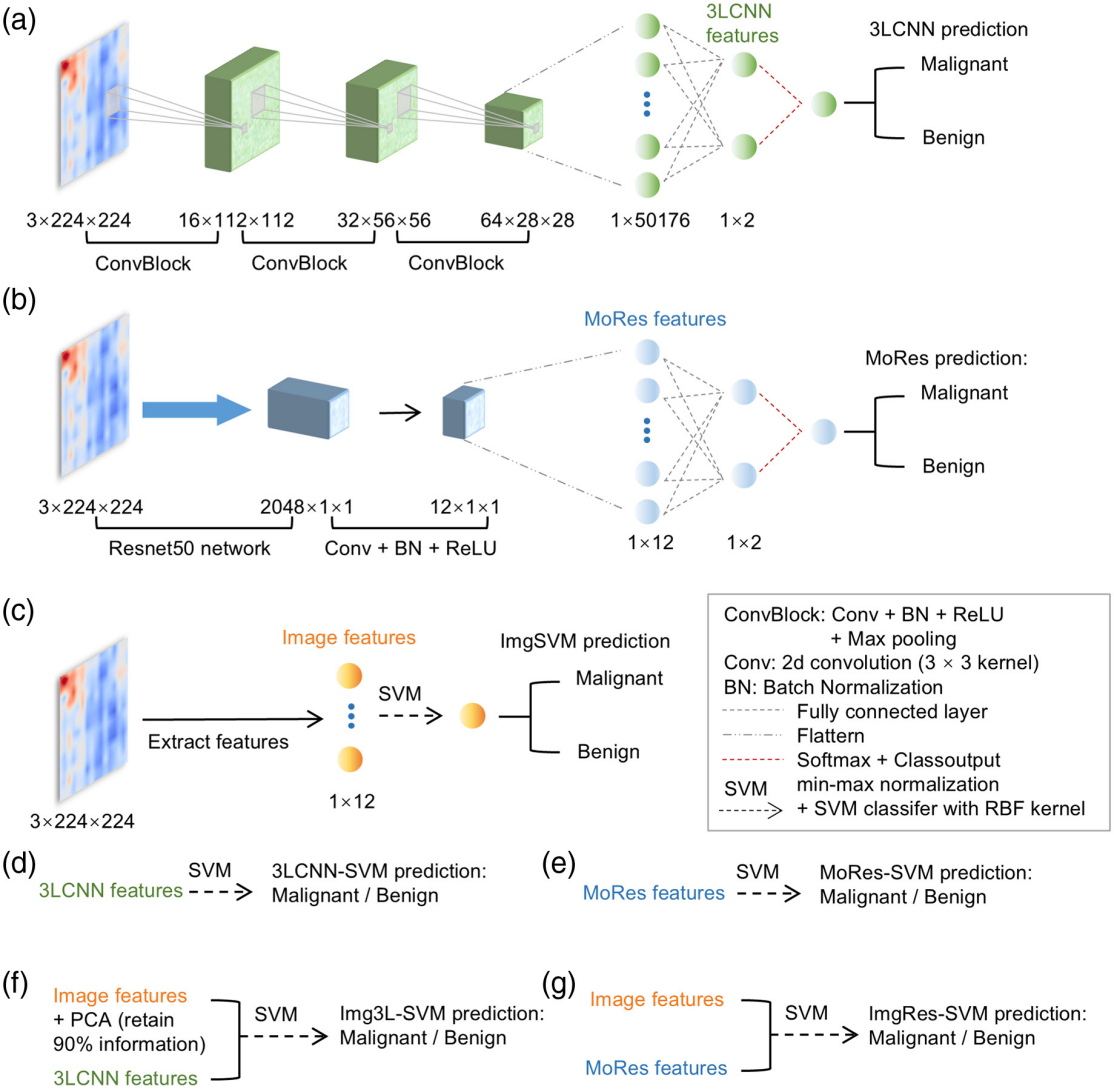
AI model schemes. (a) 3LCNN model. (b) MoRes model. (c) ImgSVM model. (d) 3LCNN-SVM model. (e) MoRes-SVM model. (f) Img3L-SVM model. (g) ImgRes-SVM model. (a)–(c) Sole models and (d)–(g) fusion models.

The DCT breast cancer diagnosis strategy (Fig. 1) was implemented by establishing three sole models, i.e., DL model (3layer CNN, 3LCNN), TL model (modified resnet50, MoRes), and ML model (SVM model based on image features, ImgSVM) as well as four fusion models, i.e., 3LCNN-SVM, MoRes-SVM, Img3L-SVM, and ImgRes-SVM.

This study primarily adopted SVM and CNN as AI-assisted diagnostic approaches. Previous studies indicated that SVM is a powerful ML model based on the theory of large margins.^45^ By constructing an optimal hyperplane for effective sample separation, SVM demonstrates exceptional classification performance and robust generalization capabilities. Furthermore, SVM is one of the most commonly used classifiers. CNN is a type of neural network designed to process data with grid-like structures, making it particularly suitable for classifying benign and malignant medical imaging data with superior performance.^45^ At present, the DCT data collected from breast clinics are scarce and not publicly available. Labeling such data requires specialized knowledge, which is also costly in both time and funds. As the first attempt at AI processing of DCT data, directly training complex DL models may encounter issues such as overfitting due to insufficient data. In addition, CNN demands substantial computational power and extended training times. By contrast, TL is frequently employed to leverage pre-trained CNN networks for more accurate classification results. The pre-trained models that are commonly used for image classification include Resnet50, VGG16, and Inception. Given that the Resnet50 effectively mitigates the vanishing gradient problem in deep networks through residual connections, meanwhile exhibiting excellent classification performance, we adopted Resnet50 as the pre-trained network for TL. Because the Resnet50 network was adopted in this study, we converted the DCT images into RGB space and subsequently resized them to match the input requirements of the Resnet50 network. To ensure consistency in model inputs (particularly utilizing images as inputs) and to maintain fair comparison between different models, the DCT image input size of the 3LCNN model was adjusted to RGB space and upsampled, to align with the input image size of the MoRes model. Moreover, fusion represents a key technique in both ML and DL, aiming at the enhancement of diagnostic performance by integrating features or models from diverse sources or levels. First, the fusion of CNN models with SVM would facilitate automatic feature extraction, along with efficient classification performance. Thus, we constructed two fusion models based on this foundation: the 3LCNN-SVM model and the MoRes-SVM model. Moreover, the features derived from a single type of image may not adequately capture the majority of relevant information. Feature fusion permits the integration of various advantages from multiple features, thereby providing AI diagnostic models with more comprehensive inputs. This integration improves the accuracy and generalization capability of AI models. Therefore, we developed two fusion models, i.e., the Img3L-SVM model and the ImgRes-SVM model, for feature fusion of DCT images.

The dataset of 59 subjects diagnosed with one lesion was divided into training and testing sets, composed of 39 and 20 subjects’ DCT images, respectively. Due to the limited availability of DCT image data, each AI diagnostic model was run repeatedly for a total of 20 times. The final evaluation parameters were averaged over these runs to achieve more stable diagnostic performance. During each running time with the same model, different subject DCT data were randomly selected for inclusion in both training and testing sets. Specifically, the training set included DCT data from 11 malignant and 28 benign cases, whereas the testing set consisted of DCT data from 10 malignant and 10 benign cases. This random division yielded 20 distinct configurations for both training and testing sets, with the aim of facilitating repeated evaluations of the AI diagnostic models. The training set employs five-fold cross-validation to reduce the issue of overfitting.

The inputs of these models consisted of the reconstructed DCT images at a size of 3×224×224 [Figs. 1(a) and 1(b)] and various features vectors [Figs. 1(c)–1(g)]. The outputs were classified into two categories: malignant and benign. In the models shown in Figs. 1(a) and 1(b), the hyper-parameters (i.e., learning rate, mini-batch size, and epochs) were optimized through five-fold cross-validation using the sparrow search algorithm (SSA),^46^ and adaptive moment estimation algorithm was used in the training stage. The number of fully connected layers was reduced to two for benign-malignant classification. In the models shown in Figs. 1(c)–1(g), the hyper-parameters (i.e., regularization parameter C and kernel function γ) were optimized through five-fold cross-validation using the grid search algorithm (GSA),^47^ and the LIBSVM package was used to implement the SVM classifier^48^ with RBF kernel. Meanwhile, default settings were applied to other hyper-parameters. The image features in Fig. 1 included the following 12 DCT features: maximum, minimum, variance, coefficient of variation, range, skewness, interquartile range, textural contrast, textural energy, entropy, full-width half maximum (FWHM) threshold, and mean rBFI greater than FWHM threshold. These 12 image features were found to statistically differentiate between malignant and benign groups in our previous study.^49^ To maintain consistency in the input feature weights of the fusion model [Fig. 1(g)], 12 MoRes features were selected from 1×12 fully connected layer. However, two 3LCNN features were selected because adding a 1×12 fully connected layer before the 1×2 fully connected layer compromised the classification performance of the 3LCNN model. Thus, to ensure consistency in input feature weights for the fusion model [Fig. 1(f)], the dimensionality of image features was reduced to 1×2 using principal component analysis (PCA).

### 2.5 Evaluation Metrics

Each classification model was replicated 20 times, and the value of evaluation metrics was averaged. The performances of those models were evaluated using a total of seven average evaluation metrics: accuracy (acc), sensitivity (sens), specificity (spec), area under the receiver operating characteristic curve (AUC-ROC), area under the precision-recall curve (AUC-PR), and the p-value of calibration curve, as well as aggregate score.

The acc was defined as the ratio of properly classified samples to the total samples. The sens is the percentage of true malignant cases in all the positive ones. The spec is the percentage of true benign cases in all the negative ones. A higher value of sens and spec indicates a lower probability of misdiagnosis. The AUC-ROC (ranges from 0 to 1) remains unaffected by the ratio of positive and negative samples. The AUC-PR (ranges from 0 to 1) exhibits more sensitivity to data imbalance and performs better when there are fewer positive class samples. A higher value of AUC-ROC and AUC-PR indicates a better model performance. In addition to discriminatory ability, all models were also tested for their calibration through the p-value of a calibration curve. The analysis was conducted using the Hosmer-Lemeshow test, a goodness-of-fit metric that compares the expected and actual probabilities within each group (groups were divided based on the predicted probability of malignancy). The model is considered to have a good fit when the p-value exceeds 0.05, indicating no significant difference between the predicted and true values. The aggregate score represents the cumulative score from the six aforementioned parameters. Briefly, the seven models are respectively sorted in descending order based on the values of these parameters, with the highest value being assigned a score of 10, and the adjacent scores being decreased by 1. In the case that multiple models share the same parameter value, they receive identical scores. As such, the maximal aggregate score would be 60.

## 3 Results

### 3.1 Repeatability of DCT Measurements on a Healthy Subject

**Fig. 2:**
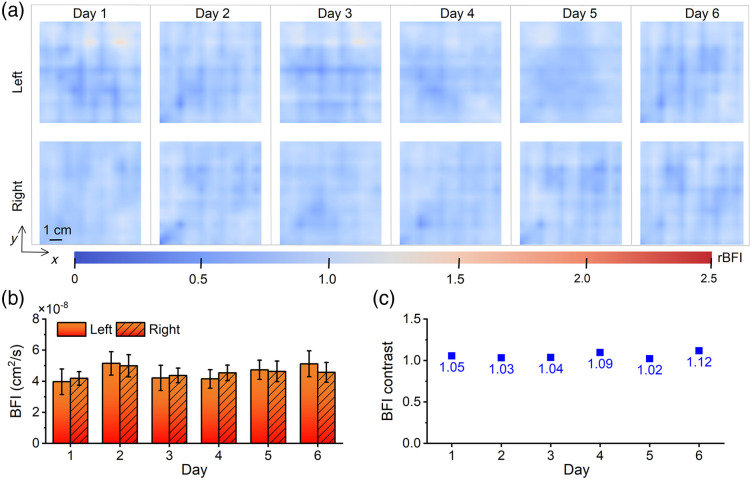
DCT measurement results of a healthy subject over 6 days. (a) Bilateral rBFI breast images. (b) Bar graph of BFI values for bilateral breasts. The bar’s height indicates the average of BFI values. Error bar indicates standard deviation. (c) The BFI contrast between bilateral breasts. The blue square dots represent the absolute value of the left-to-right breast BFI ratio.

A healthy female subject was recruited to receive daily DCT measurements of left and right breasts at 9 am for six consecutive days. To ensure consistent measurement positioning, probe placement was marked before the initial assessment. Figure 2(a) illustrates the DCT image outcomes. The rBFI of DCT breast images exhibited relatively uniform distribution. Bilateral breast rBFI demonstrated higher similarity, whereas unilateral DCT breast images exhibited elevated rBFI in corresponding regions. The variability of repeat measurements on the same subject was observed to be low, as depicted in Fig. 2(a); hence, the non-normalized BFI data were presented in Figs. 2(b) and 2(c). The mean BFI of the subjects’ breasts remained relatively stable around $4.5\times 10^{-8}\;{\mathrm{cm}}^{2}/s$, with the left breast slightly smaller than the right breast. The BFI contrast (absolute value of ratio) in both breasts ranged from 1.02 to 1.12, indicating a small variability and stable measurements.

### 3.2 Representative DCT Images with Malignant and Benign Lesion

**Fig. 3:**
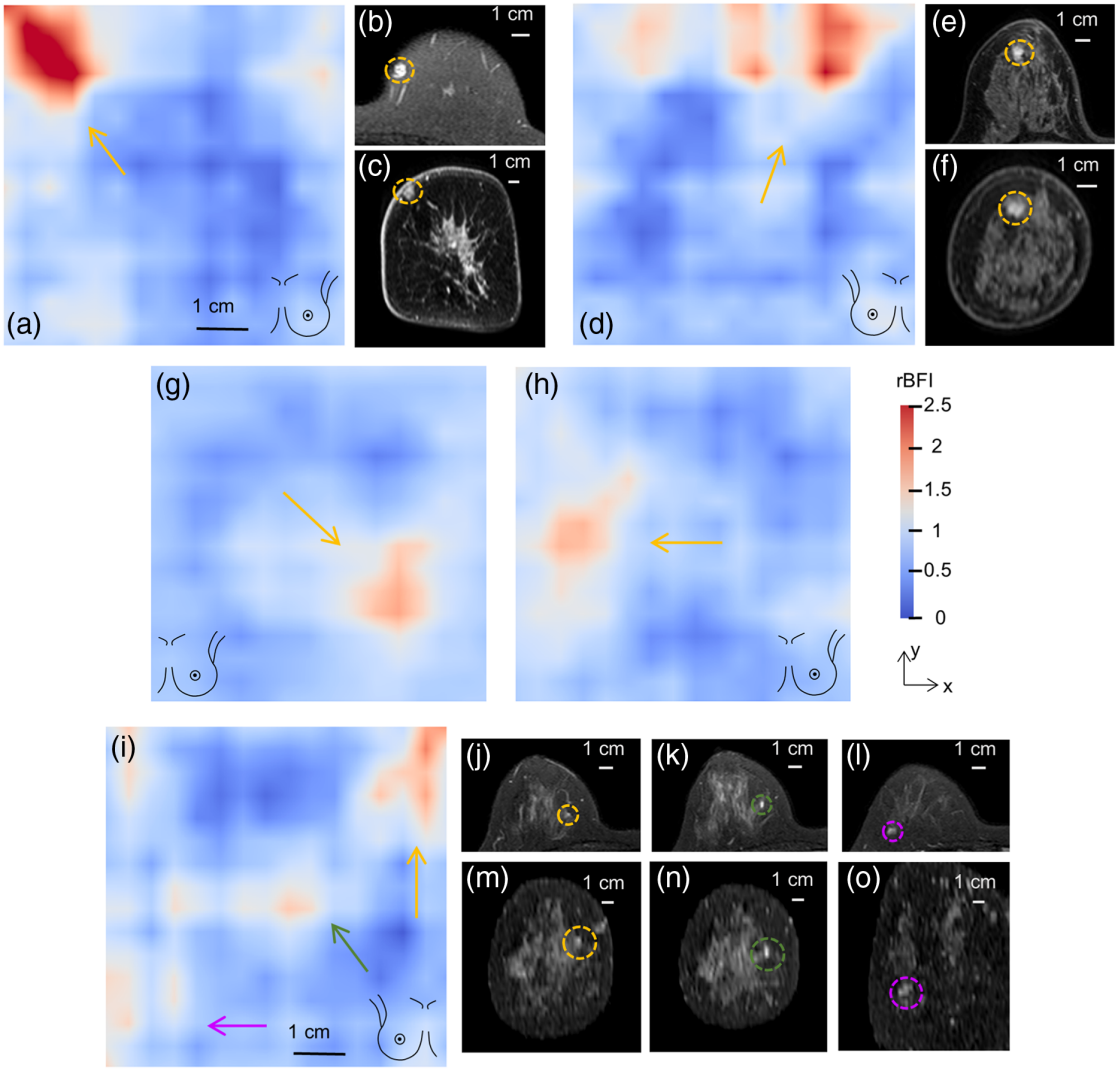
Representative DCT and MRI images that contain lesions. (a) A malignant lesion appeared on the upper left quadrant and corresponded to the presence of a nodular long T2 signal shadow observed in the MRI images with (b) foot-to-head orientation and (c) anterior-to-posterior orientation, also located within the upper left quadrant of the breast. The higher rBFI exhibited a larger extent of the region when compared with the region of nodular long T2 signal shadow (measuring 0.80  cm×0.84  cm in size). (d) A malignant lesion indicating small differences between DCT and MRI images. The nodular high T2 signal shadow was observed in the MRI images and appeared within the (e), (f) upper left quadrant (about 11 o’clock, measuring 0.87  cm×0.98  cm in size) of the right breast, whereas the high blood region was observed on the upper right quadrant. (g) A benign lesion (cyst) appeared around 3 to 4 o’clock and corresponded to the high blood region of the left breast. (h) A benign lesion (fibroadenoma) appeared on the upper quadrant, whereas the high blood region was observed on the left quadrant. (i) Three benign lesions were located in the upper right, middle, and lower left, respectively. These regions closely corresponded to the presence of three nodular high T2 signals observed in the MRI images and appeared within the (j), (m) inner upper quadrant, (k), (n) inner quadrant, and (l), (o) outer lower quadrant of the right breast, respectively. The high rBFI also exhibited a larger extent of region when compared with the region of nodular high T2 signals, with the largest lesion being measured as 0.69  cm×0.56  cm.

The DCT images with malignant lesions and benign lesions are presented in Fig. 3 as representative cases. Compared with the morphological changes of tumors in clinical diagnostics, blood flow abnormalities exhibit a broader extent, indicating that alterations in tumor microenvironmental blood flow may precede morphological changes.

Overall, the lesion location on DCT images from 39 women was found to be consistent with clinical diagnostics [as shown in Figs. 3(a), 3(g), and 3(i)], whereas the outcomes from 21 women exhibit small differences due to position deviations [as shown in Figs. 3(d) and 3(h)]. The detection of blood flow abnormalities in breast lesions through DCT measurements might be influenced by the ROI selections and thereby inherent differences with morphological structure (MRI, ultrasound). In the DCT images of 60 subjects with lesions, larger rBFI magnitude and heterogeneity were generally observed in malignant lesions when compared with benign lesions. Therefore, DCT measurements demonstrate the capability to distinguish between benign and malignant lesions in women’s breasts.

The MRI images in Fig. 3 primarily illustrate lesion type and location, not for quantitative comparison between MRI and DCT. Precise comparison requires concurrent measurements and spatial registration, which will be the goal of our future research.

### 3.3 Performance of AI Models for Differentiating Benign and Malignant Lesions

**Fig. 4:**
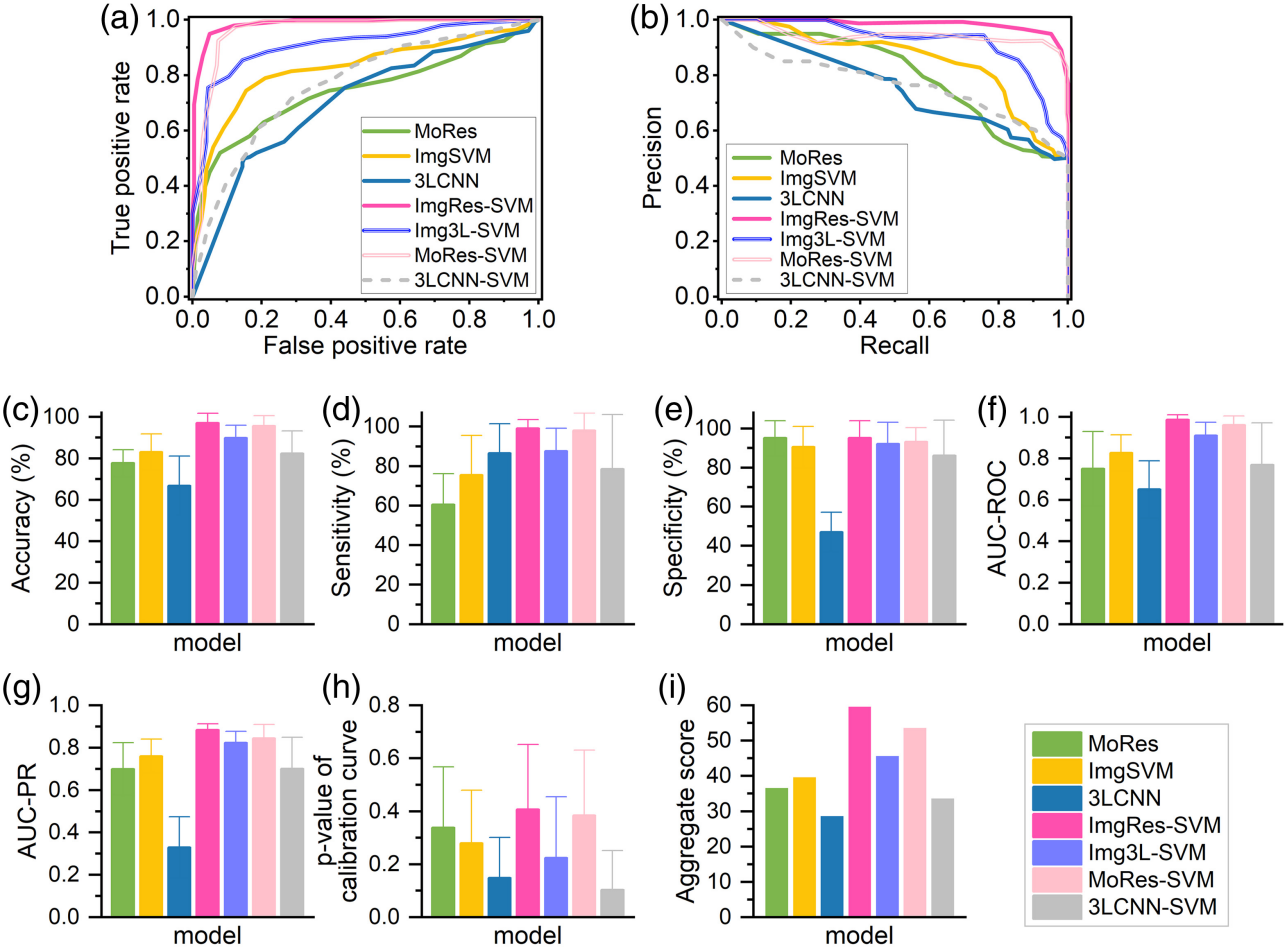
Performance of AI models in classifying benign and malignant lesions. (a) ROC curves. (b) PR curves. (c)–(i) Bar graphs of other evaluation metrics. The bar’s height indicates the average of metrics through 20-time replications. Error bar indicates standard deviation.

The performance of benign-malignant classification models is illustrated in Fig. 4 and Table 1 through a comparative analysis of evaluation metrics.

**Table 1:** Comparison of AI diagnostic models.

AI model	Performance	Advantage
ImgRes-SVM model	Aggregate score of 60 and accuracy of 97%	Exhibiting top-performance in the classification of benign and malignant cases through fusion
MoRes-SVM model	Aggregate score of 54 and accuracy of 95.5%	Improving classification performance by solely using the MoRes model
MoRes model	Aggregate score of 37 and accuracy of 77.5%	Permitting automatic feature extraction
Img3L-SVM model	Aggregate score of 46 and accuracy of 89.75%	Exhibiting excellent performance in the classification through fusion
3LCNN-SVM model	Aggregate score of 34 and accuracy of 82.25%	Improving classification performance by solely using the 3LCNN model
3LCNN model	Aggregate score of 29 and accuracy of 66.75%	Permitting automatic feature extraction
ImgSVM model	Aggregate score of 40 and accuracy of 83%	The combination of manually extracted image features and SVM showed better performance.

In general, the performance of the fusion model is better than that of the sole model. The ImgRes-SVM model demonstrates the best performance in categorizing breast lesions as either malignant or benign, with the highest aggregate score of 60, an average accuracy of 97%, sensitivity of 99%, specificity of 95%, AUC-ROC of 0.986 (95% confidence interval, CI 0.937 to 1), AUC-PR of 0.885 (95% CI 0.829 to 0.939), and p-value of 0.407. The MoRes-SVM model achieves an aggregate score of 54, average accuracy of 95.5%, sensitivity of 98%, specificity of 93%, AUC-ROC of 0.960 (95% CI 0.874 to 1), AUC-PR of 0.845 (95% CI 0.717 to 0.972), and p-value of 0.385. The Img3L-SVM model achieved an aggregate score of 46, average accuracy of 89.75%, sensitivity of 87.5%, specificity of 92%, AUC-ROC of 0.909 (95% CI 0.782 to 1), AUC-PR of 0.823 (95% CI 0.717 to 0.929), and p-value of 0.224. The ImgSVM model achieved an aggregate score of 40, average accuracy of 83%, sensitivity of 75.5%, specificity of 90.5%, AUC-ROC of 0.826 (95% CI 0.654 to 0.998), AUC-PR of 0.756 (95% CI 0.597 to 0.915), and p-value of 0.28. The MoRes model achieved an aggregate score of 37, average accuracy of 77.5%, sensitivity of 60.5%, specificity of 95%, AUC-ROC of 0.750 (95% CI 0.567 to 0.934), AUC-PR of 0.700 (95% CI 0.578 to 0.824), and p-value of 0.34. The 3LCNN-SVM model achieved an aggregate score of 34, average accuracy of 82.25%, sensitivity of 78.5%, specificity of 86%, AUC-ROC of 0.768 (95% CI 0.369 to 1), AUC-PR of 0.701 (95% CI 0.413 to 0.990), and p-value of 0.104.

**Fig. 5:**
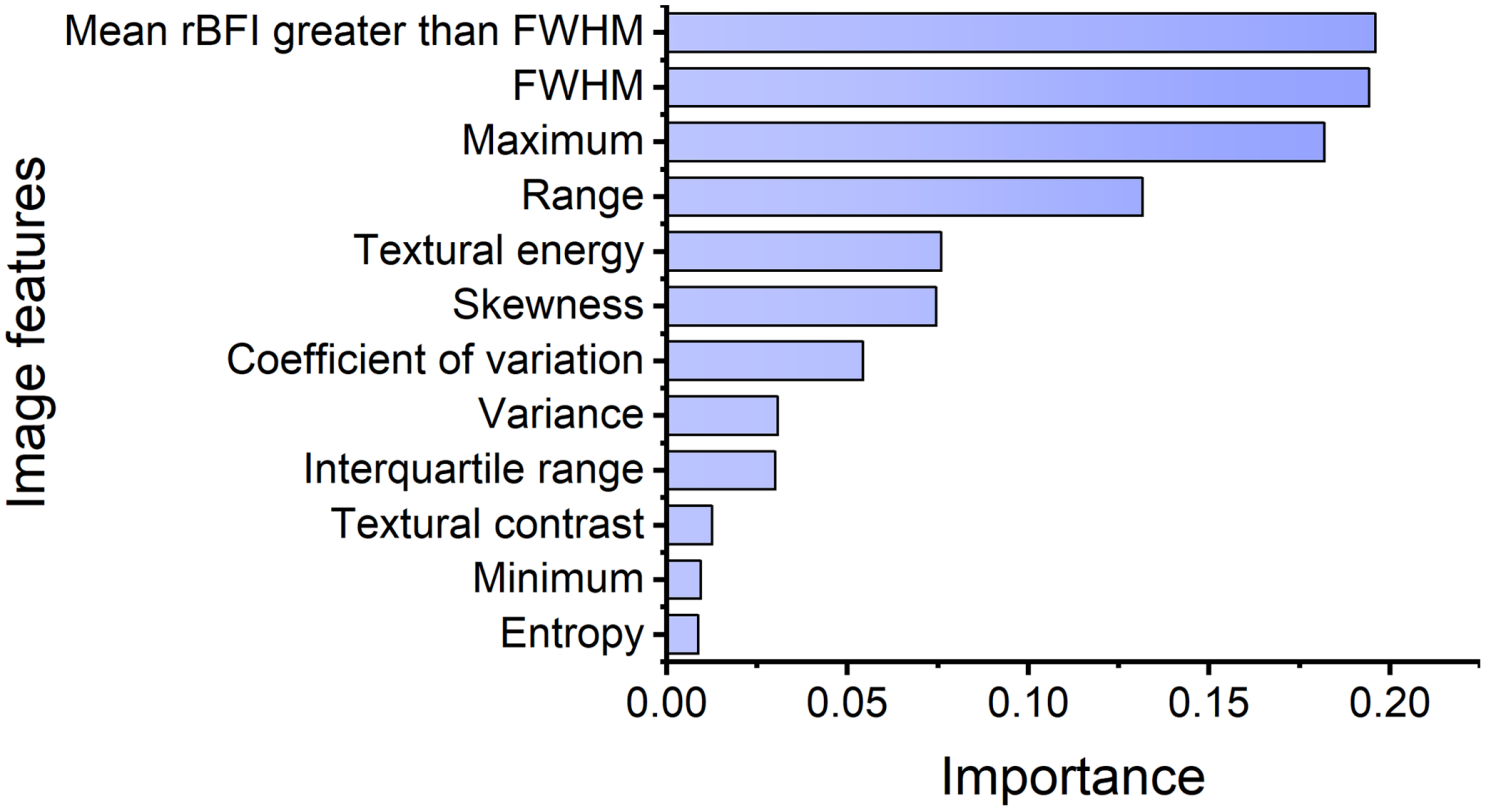
Importance of image features.

To assess and visualize the importance of each image feature utilized as input for AI models, we adopted the random forest to calculate feature importance for all image features, as illustrated in Fig. 5. According to the random forest algorithm, the importance of each feature is defined as the average information gain attributed solely to that feature across all trees. The calculated importance values are normalized, so that their sum is equal to 1. The higher value of importance indicates that this image feature (e.g., the mean rBFI greater than the FWHM threshold) is more important than others.

**Fig. 6:**
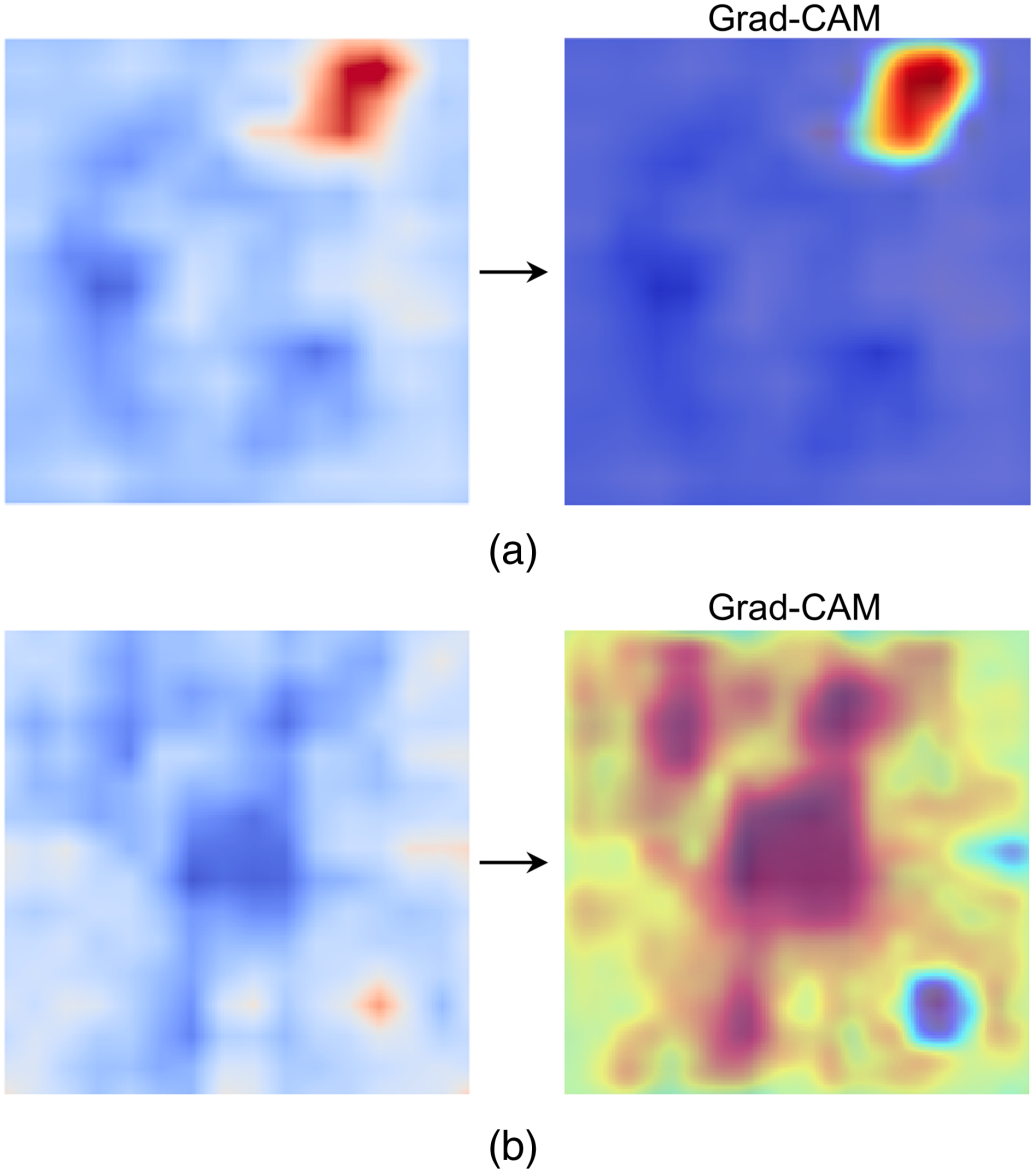
Heat maps that were calculated using Grad-CAM of neural network for (a) a malignant lesion and (b) a benign lesion, both classified by the 3LCNN model.

To further understand the features extracted by neural networks and assess where the neural network pays more attention, we applied Grad-CAM,^50^ an approach for visual explanations from deep networks through gradient-based localization, to the reconstructed BFI images. Grad-CAM calculates the backward gradient based on the final prediction and generates a heat map that can be directly overlaid on the original DCT image. This visualization highlights both the areas of interest for the neural network and those regions that contribute more to the final prediction. Taking the 3LCNN model as an example, Fig. 6 shows the results of one malignant and one benign case that were derived from Grad-CAM. The heat map corresponding to the malignant lesion emphasizes the regions with high blood flow, whereas the heat map for the benign lesion predominantly focuses on the regions exhibiting normal blood flow. The 3LCNN model exhibits limited attention to regions of elevated blood flow within DCT images of benign lesions, which might lead to inaccuracies in predictive outcomes. By integrating the 3LCNN features with image features, the fusion model can place greater emphasis on both normal blood flow regions and those with locally elevated perfusion, thereby enhancing the diagnostic performance of the model.

## 4 Discussion

In this study, we demonstrated several innovations of a custom-designed DCT system for breast tumor identification in the following aspects. (1) In the instrumentation construction, the fast and stable data acquisition over the breast was achieved through the design of a concentric circle distribution mode in an S-D array and the utilization of an optical switch. As a consequence, the DCT data acquisition from a clinical setting was stable and less affected by ambient noise, when compared with the conventional spatial scanning method. This paradigm also greatly reduces the measurement time, minimizes motion artifacts, and helps maintain comfort measurement posture for subjects. (2) The NL-DCT imaging framework improved the accuracy and robustness of DCT image reconstruction by taking into account information on light propagation within the breast tissues, which revolutionarily differs from the conventional analytical solution or finite element method that seeks for the solution of partial differential equation. (3) The integration of our DCT hardware system architecture and reconstruction algorithm facilitated the acquisition of high-quality breast data from a total of 59 subjects. This is the first pilot investigation for DCT breast lesion classification through the fusion of AI strategy and DCT technology. (4) The longitudinal monitoring of healthy subjects has demonstrated the stability of the DCT measurements that were also applied to the patients with breast tumors. (5) This study evaluated seven proposed AI diagnostic models. We found that the ImgRes-SVM model achieved a more accurate diagnosis of breast cancer based on a certain number of DCT datasets (including 59 subjects). This finding indicates the feasibility of combining DCT and AI models for future clinical applications in identifying breast cancer.

The DCT technique identifies the malignant degree of breast lesions by investigating the scattering movement of red blood cells, which reflects the increased hemodynamics and metabolism resulting from breast tumor division and proliferation. On the other hand, conventional first-line diagnostic investigations, such as mammography, ultrasound, and MRI, identify the malignant degree of breast lesions according to the substantial morphological changes of breast tumors. These conventional breast cancer diagnosis techniques have been developed in maturity. The AI-assisted DCT proposed in this paper is an exploration of combining AI and DCT for breast cancer diagnosis, although its accuracy and stability might not be as perfect as conventional first-line diagnostic techniques. However, the relationship between breast lesion hemodynamics, metabolic information, and physiological pathology would exhibit a more direct correlation, thereby serving as a fundamental basis for future research. The purpose of this study is not to replace conventional breast cancer diagnosis techniques but to establish a link between microcirculation networks (i.e., red blood cell movement) and macro-malignant degree of breast lesion. The AI-assisted DCT is a valuable supplement to conventional morphological diagnostic techniques. The combination of functional and morphological techniques has the potential to yield a breast cancer biomarker in future research.

Breast tumor angiogenesis can be robustly explored by microvascular blood flow, which is directly related to tumor proliferation and vessel abnormalities caused by cancellous cells. Previous case studies have quantified the BFI of breast lesions in the microcirculatory network and demonstrated that malignant lesions typically show relatively higher BFI in comparison with benign lesions.^34^^,^^38^ This study exhibited consistent outcomes compared with previous findings. However, blood flow has not been investigated to a similar degree to categorizing the benign and malignant breast lesions using AI models within a relatively large population, due to the difficulties in instrumentation, imaging strategies, and the appropriate approaches for imaging analyses. These outcomes derived from 61 subjects demonstrated the capability of AI-assisted DCT for identifying breast cancer automatically.

AI models performed differently in discriminating malignant and benign lesions depending on the options of input features and classification algorithms. The input features of models were selected through diverse methods, thereby leading to varied degrees of classification outcomes. As for the classification algorithm, the fusion models have better performance than the sole models, whereas the ImgSVM appeared as the best sole model.^28^^,^^45^ In terms of the 3LCNN classification strategy, we selected the training from scratch because the DCT dataset exhibits remarkably distributional differences from large-scale datasets (e.g., ImageNet). Fine-tuning a pretrained model might introduce irrelevant features. The parameters of each layer in the 3LCNN model were entirely derived from DCT image data during the training process from scratch, which aligns more closely with the classification requirements for DCT breast lesion detection. Due to the less complicated three-layer architecture, we hypothesized that training from scratch could avoid overfitting, meanwhile maintaining computational efficiency. Furthermore, the 3LCNN model has a relatively shallow architecture, which facilitates the visualization of its feature heat maps (i.e., Fig. 6). This characteristic enhances our understanding of how the model extracts features from raw data, thereby improving the interpretability of DCT classification.

Among AI models that we have investigated based on 59 subjects, the ImgRes-SVM model demonstrated the best performance in breast lesion classification. In comparison to other publicly available extensive datasets for breast lesions, such as those from ultrasound and MRI,^45^ this study utilized the relatively limited DCT image dataset (including 59 subjects). It can be anticipated that when the number of DCT data increases, the performance of AI diagnostic models might further improve. Our classification study from a limited dataset provides a research basis for future larger-scale diagnosis of breast lesions using DCT. In the MoRes-SVM model, the integration of image features and MoRes features was crucial in enhancing the performance of the model. The image features provided interpretability and clues for distinguishing between malignant and benign lesions, based on local perfusion of microvascular blood flow (represented by the following image features: maximum, minimum, FWHM threshold, mean rBFI greater than the FWHM threshold) as well as blood flow heterogeneity (characterized by the following image features: variance, coefficient of variation, range, skewness, interquartile range, textural contrast, textural energy, entropy). Adding MoRes features could effectively improve the efficiency of classification. Feature fusion is a synergistic method that takes into account the interpretability and accuracy of models.

The benign-malignant outcomes derived from the ImgRes-SVM model included false positives and false negatives. The DCT image of the false positive case generally exhibited higher rBFI, leading to a misclassification as malignant, which would be attributed to endocrine disruption or accelerated growth rate of some benign lesion (with fibroadenoma^51^ as an example). Similarly, the DCT image of false negatives case generally exhibited lower rBFI, which would be attributed to slow growth, low microvessel density, and reduced structural complexity of some malignant lesions (with invasive ductal carcinoma Nottingham grade II^52^ as an example). These misclassifications, which imply more physiologic reasons, will be one subject of our future research.

A few factors may account for the discrepancy between the well-classified results presented in Fig. 4 and the situation where some high blood flow regions do not exactly match the clinical diagnostic outcomes (shown in Fig. 3). First, the difference between the high blood flow regions and clinical diagnostic outcomes might be attributed to the measurement posture (supine for DCT versus prone for MRI) and diagnostic principle (hemodynamic functional information of breast lesion for DCT versus morphological structures for MRI and ultrasound). Furthermore, the DCT data acquisitions were performed by breast specialists based on their clinical diagnostic experiences. During this process, the probe was positioned over breast surface areas of interest (ROI) containing lesions. The custom-designed optical probe used in this study covers an area measuring $8\times 8\;{\mathrm{cm}}^{2}$. Thus, the average maximal tumor sizes observed in this study (benign group: 11.4±8.4  mm; malignant group: 20.4±9.7  mm) are remarkably smaller than this coverage area, ensuring that the lesion was included within the DCT image. Although there exist some position deviations between the lesion and high blood flow regions on the DCT image, it is safe to conclude that the DCT image represents the imaging outcomes related to the lesion. To preclude the combined effects from multiple lesions, only subjects with one lesion were included in our classification study. Note that the DCT images from subjects with three benign lesions are intended solely to illustrate the consistency between high blood flow regions and corresponding MRI diagnostic positions of lesions but are not used for lesion classification by AI models.

The tomographic images of BFI, rather than $g_{1}(\tau )$ curves, were adopted as the first attempt to classify the malignant and benign tumors in this study. As the raw data of DCT, the $g_{1}(\tau )$ curves, representing information of the light electric field, are not directly related to the blood flow anomaly caused by breast cancers. For example, an elevation of blood flow within the local region generally indicates malignant tumors when compared with the surrounding normal tissues, which could be clearly visualized from the tomographic BFI images, but hardly identified by the raw $g_{1}(\tau )$ curves. In addition, the NL-DCT algorithm that was developed in our laboratory is capable of incorporating the information of photon migration within each voxel for image reconstruction, yielding more accurate BFI imaging than the conventional DCT image reconstruction algorithms such as analytical solution or finite element method. In addition, the application of the Bregman-TV method to the NL-DCT algorithm significantly enhanced the uniformity of blood flow in non-marginal regions. Therefore, the tomographic DCT images reflect more breast physiological status, when compared with the original light electric field data (i.e., $g_{1}(\tau )$ curves). Inspired by the efforts that identify the disease lesions from the raw ultrasound data (i.e., radio frequency time series),^53^ we will explore the feasibility of using the raw DCT data ($g_{1}(\tau )$ curves) to identify breast cancers via AI models in future studies.

Please note that DCT image reconstruction was performed in a 3D space, resulting in tomographic images along the penetration depth (i.e., z-axis). As a result, the three-dimensional DCT images were generated on a geometrical model of the $8\times 8\times 3\;{\mathrm{cm}}^{3}$ at the size of 16×16×6 (i.e., the voxel size is $0.5\times 0.5\times 0.5\;{\mathrm{cm}}^{3}$). The pixel size of the two-dimensional DCT image utilized for image classification in this study was 16×16, located at z=2, corresponding to a depth range of 5 to 10 mm beneath the surface of the breast tissue. The outcomes derived from our previous phantom experiments reveal that the 2D image at this depth range is most accurate within this volume.^42^ Other studies also suggested that deeper depth exhibits lower reconstruction accuracy.^38^ Therefore, we selected 2D DCT images at the depth range of 5 to 10 mm, instead of the entire 3D images, for classification. Further improvement of the BFI image reconstruction by the advanced approaches (e.g., DL framework), particularly in-depth beyond 10 mm, is one subject of our future works.

Note that most of the subjects participating in this study did not receive MRI imaging, which could provide the geometrical information of the breasts. For the purpose of fair comparisons under a consistent setup, the photon propagation was performed on a standard volume (at the size of $8\times 8\times 3\;{\mathrm{cm}}^{3}$), to match the size of an optical probe (i.e., $8\times 8\;{\mathrm{cm}}^{2}$) for breast measurement at the penetration depth no more than 3 cm. During the optical measurements, we carefully monitored the light signals and ensured there was no mismatch between the optical probe and breast tissue, even if there were shape differences among individual breasts. Future efforts will be made to incorporate the geometrical information (derived from CT or MRI) into visualized Monte Carlo software (i.e., MCX^54^), to enhance the accuracy of BFI imaging over breast.

The optical properties (e.g., scattering and absorption coefficients) were assumed to be constant across all subjects in this study because the scattering and absorption coefficients were not measured on those subjects. The image reconstruction would be influenced by those optical properties. According to previous studies,^36^^,^^38^ the blood flow values on malignant tumors are often more than 10-fold higher than the surrounding normal tissues, much higher than the variability of absorption coefficient and reduced scattering coefficient (generally no more than two-fold contrast). Hence, the influence of optical properties on BFI image reconstruction could be considered to be minor. Nevertheless, precise reconstruction of the BFI would be achieved from the simultaneous incorporation of tissue optical properties (primarily, absorption coefficient and the reduced scattering coefficient) using the frequency-domain or time-domain oximeter,^55^ which is one subject of our future research.

Because the DCT data collected from breast clinics are scarce and not publicly available, the number of DCT images used as input for AI diagnostic models is limited when compared with the routine clinical techniques (e.g., ultrasound, MRI), which could lead to biased model performance. Prior to the AI diagnosis, every effort was made to reduce the limitation of a dataset, such as the implementation of random partitioning of the training and testing datasets, performing the repeated training of the same model multiple times, and applying cross-validation techniques. Moreover, we employed pre-trained networks (Resnet50) and fusion methods to reduce the concern arising from insufficient datasets.

AI-assisted DCT has demonstrated great potential for breast cancer diagnosis. Future studies will further explore the fusion techniques, such as the combination of DCT with MRI, aiming to develop more comprehensive and multimodal diagnostic approaches for breast cancer. The integration of DCT with concurrent clinical MRI for breast imaging could facilitate the simultaneous detection of hemodynamic functional information and morphological structural details of lesions, thereby permitting a complete assessment of breast lesion physiology. Furthermore, MRI provides precise spatial localization, which could be used as a prior to improve the accuracy and robustness of DCT image reconstruction.

This study is also subject to several limitations. Although it is a successful DCT measurement on a relatively large group of women with breast tumors, the sample size was still small compared with other well-established imaging diagnostic methods for breast cancer, and therefore, the different types of lesions were generally categorized into benign and malignant groups without considering the distinctions among subtypes. Besides, some lesions investigated in this study did not have tissue diagnosis (i.e., biopsy testing), but were characterized as clearly malignant on MRI by experienced radiologists. Future studies will expand our data collection over a larger population and further refine the AI model to enhance its clinical precision for early diagnosis of breast cancer. The spatial regularization methods (e.g., ultrasound-guided DCT) were not employed in this study to ensure accurate spatial localization of the tumor. In future studies, the same measurement posture makes DCT highly compatible with conventional first-line diagnostic investigations and ultrasound, thereby enabling the simultaneous assessment of microvascular blood flow and tumor morphology detection. The utilization of an optical fiber probe facilitates DCT integrated with DOS/DOT, which can provide comprehensive insights into the neoangiogenesis and oxygen metabolism of tumor growth. Multimodal imaging enables the detection of multidimensional tumor information while minimizing the risks associated with misdiagnosis. This study provides experimental support for the combination of functional information and morphological information.

Apart from the aim of lesion identification, the AI-assisted DCT system also has the potential to be implemented in clinical settings for therapeutical assessments, such as predicting response to neoadjuvant chemotherapy as well as investigating breast tumor-induced angiogenesis or alterations in the microcirculatory network. In the future, we hope that microvascular blood flow biomarkers for cancellous cells will be derived from the bulk DCT measurements combined with AI models.

## 5 Conclusions

We presented an AI-assisted DCT system for breast cancer diagnosis based on microvascular blood flow tomographic images. For this purpose, a DCT system incorporating unique imaging strategies was constructed for breast tissue measurements. The use of a specific probe and optical switch allows for rapid and efficient collection of DCT signals with minimal motion artifacts. Those advances in DCT instrumentation allow us to collect high-quality photon signals from a total of 61 women for reconstructions of blood flow tomographic images and identification with AI models. The DCT system has demonstrated remarkable advantages in measurement stability (bilateral BFI contrast in healthy subjects exhibited a slight range of 1.02 to 1.12) and breast cancer risk assessment (distinguishing benign and malignant lesions with an accuracy of 97%). By comprehensive comparisons, we identified an AI model with excellent classification performance among a total of seven proposed AI diagnostic models for breast cancer diagnosis, based on a DCT image dataset containing a total of 59 subjects. As DCT is an emerging functional imaging modality, bulk data accumulations and proper selection of AI models would greatly enhance its capacity to improve the accuracy, sensitivity, and specificity of breast cancer diagnosis, and support the rapid translation of DCT technology into routine clinical usage.

## Supplementary Material

10.1117/1.JBO.30.5.055001.s01

## Data Availability

Data underlying the results presented in this paper are not publicly available at this time but may be obtained from the authors upon reasonable request.
