# Supplementary material for: AI-assisted diffuse correlation tomography for identifying breast cancer
Source: J Biomed Opt. 2025 May 16;30(5):055001. doi: 10.1117/1.JBO.30.5.055001 (PMC12083502; doi:10.1117/1.JBO.30.5.055001)
Supplement: Supplementary file 1 [file JBO_030_055001_SD001.pdf]

Supplemental materials for article entitled, “**Artificial intelligence assisted diffuse correlation tomography for identifying breast cancer**”

Supplemental materials are used to elucidate the instrumentation setup and measurement procedure of custom-designed DCT system. The custom-designed DCT system constructed in our laboratory is shown in Fig. S1(a), with schematic diagram illustrated in Fig. S1(b). Briefly, the system consists of a NIR light emission/reception module, an extended field of view (FOV) module, a fiber-optic probe module and a data processing module.

The DCT device was equipped with a long-coherence (> 5 m) laser (785 nm, DL-785-120-SO, Crystallaser Inc., USA), six single-photon detectors (SPCM-780-13-FC, Excelitas Inc., Canada) and an eight-channel digital correlator (Flex19OEM-8ch, www.correlator.com, USA) as the NIR light emission/reception module. The laser emits light at an output power of 70mW over the tissue interface, without inducing any adverse effects on breast tissue, such as invasion and radiation. The single-photon detectors with avalanche photodiode provides a vital improvement in signal-to-noise ratio (SNR) for detecting fluctuations of laser speckle compared to conventional photomultiplier tube.

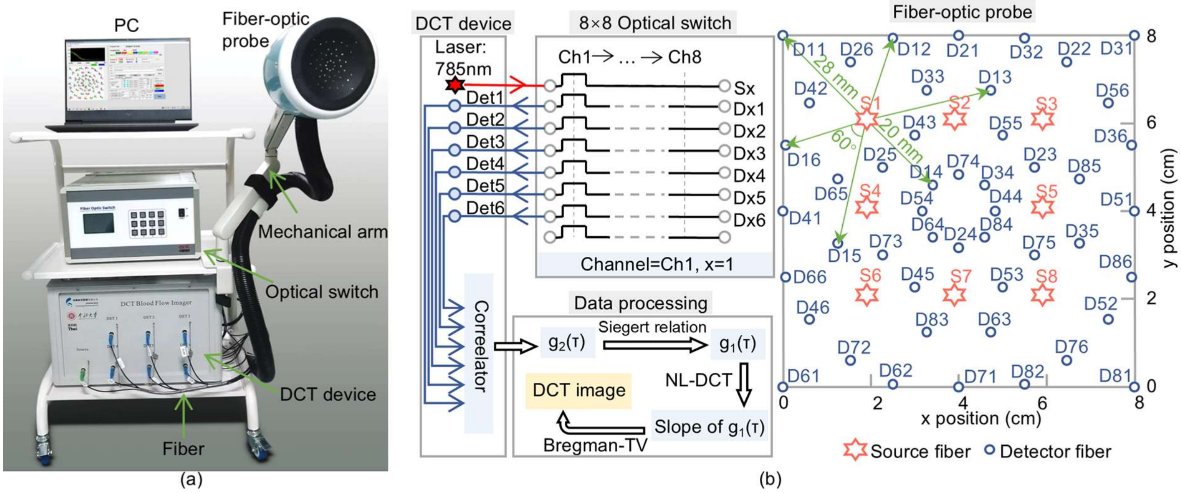

Fig. S1. DCT system. (a) DCT instrumentation setup. The connection of DCT device, optical switch, and probe is implemented through the utilization of optical fibers. (b) Schematic diagram of DCT system for blood flow imaging. Detj represents jth detector (j = 1, …, 6, detector number). A S-D fiber set (chx, x = 1, …, 8, channel number) consisted of one source fiber (Si, i = 1, …, 8, source fiber number, denoted by the asterisk) and six detector fibers (Dij, j = 1, …, 6, detector fiber number, denoted by the circle). i = 1, 3, 6, 8, a large 2.83 cm for Si-Di1, Si-Di3, Si-Di5, a short 2 cm for Si-Di2, Si-Di4, Si-Di6; i = 2, 4, 5, 7, a short 2 cm for Si-Di1, Si-Di3, Si-Di5, a large 2.83 cm for Si-Di2, Si-Di4, Si-Di6. The status of optical switch depicted in Fig. S1(b) currently indicates the operation of channel 1, wherein the optical switch is connected to S1, D11, D12, D13, D14, D15, and D16 on the fiber-optical probe. The DCT data acquisition is completed after transitioning from ch1 to ch8. The breast surface is formed by x-y axis. The z-axis is perpendicular to the breast surface, i.e., anterior-to-posterior direction of the female subject. The DCT images presented in this article are situated at a depth of z = 1 cm.

Due to the high cost of laser and detectors in the DCT system, an 8 × 8 channel optical switch was designed as extended FOV module to permit cost-effective integration of a large number of source and detector positions. This module facilitated extension from source-detector (S-D) pairs [consisting of a laser and six detectors, as shown on the left side of Fig. S1(b)] onto S-D fiber set in the probe [illustrated on the right side of Fig. S1(b)]. A specific set of S-D fiber pairs (consisting of 6 S-D fiber pairs) was sequentially switched from ch1 to ch8 at intervals of 15 seconds, taking a total of 2 minutes for a measurement containing 48 S-D fiber pairs (i.e., 6 × 8). The sequence of

photon transmission is as follows: laser, source fiber, breast tissue, detector fiber, and the detector. In comparison to conventional handheld measurements (which involves manually moving a grid-array probe in a straight way), the use of this extended FOV module (with the probe controlled by mechanical arm) simplifies the operation, reduces measurement time and ensures the accurate sensor localization, making it more suitable for clinical breast cancer detection. Compared with the noncontact DCT measurements, which involve a rotate line-shape probe scanning over region of interest (ROI), our DCT system is equipped with the extended FOV module, providing the advantage of easy installation and insensitivity to ambient light and motion artifacts. These advantages make it more feasible for obtaining high-quality BFI image reconstruction, thereby facilitating accurate clinical quantification of breast tumors.

The fiber-optic probe module consists of blue outer shell, white fiber-optic fixed base and black soft adhesive patch [shown in top right of Fig. S1(a)]. The outer shell and fiber-optic fixed base were utilized to ensure the protection and integrity of the fiber during breast measurement. The black soft adhesive patch, covered within fiber-optic fixed base, is utilized to ensure the comfort and safety during usage, meanwhile maintaining close fitness between the probe and breast tissue. This design effectively minimizes the influence of ambient light on measurements. The rear end of the probe was connected to a mechanical arm, which permits the probe to be freely moved and rotated until a proper contact with breast surface is identified. The reconstructed images of DCT in the breast are strongly dependent on the distribution of S-D array, which also determines the covering ROI and penetration depth. For example, a longer S-D distance leads to a larger penetration depth beneath the tissue surface, but is subjected to a lower SNR. The shorter S-D distance exhibits a limited penetration depth of blood flow measurement, along with relatively higher SNR signals. To ensure a balanced photon transmission over the tissue ROI, a unique S-D array was designed to cover a sufficiently large area [8×8 cm^2, shown in the right side of Fig. S1(b)] by using the limited number of S-D pairs. The probe comprises a total of 48 S-D fiber pairs, with each set consisting of 6 S-D fiber pairs positioned at an angle of 60° between the adjacent pair. The distances between the source and detector were cross-aligned over each S-D set, with a longer distance at 2.83 cm and a shorter distance at 2 cm. The S-D array designed in this study demonstrates several emerging features such as the circled covering ROI, balanced photon detections and symmetrical distribution. This kind of S-D array maximizes the detection area and enhances the robustness of optical detection.

The procedures for DCT measurement with our custom-designed DCT system were depicted as follows:

(1) The subject lied in a supine position on the hospital bed, and was instructed to maintain still and breathe normally. The right breast and the surface of the probe were both cleansed by using alcohol prep pads.

(2) The DCT instrument operator used a mechanical arm to manipulate the contact of probe with the breast ROI, ensuring a minimal compression to the breast tissue. Additionally, the movement of the probe was real-time adjusted based on subject feedback.

(3) Before formal measurement, the operator performed a 24-second scan over the breast (3-second scanning for each channel), meanwhile closely monitoring the fluctuations in the $g_1(\tau)$ function to ensure optimal probe-breast contacts. In the case that abnormal contact was detected, the operator promptly adjusted probe position.

(4) Automatic measurement: the source laser of DCT probe were sequentially excited via computer control panel, and the detector array simultaneously collected spatial fluctuations of light

speckles on the tissue surface. The complete measurement on the target breast lasted 2 minutes (i.e., 15 seconds scanning for each channel).

(5) After the measurement, the optical probe was removed from right breast and placed on left breast after being cleaned with alcohol prep pads. The Step (2) to (4) were repeated until the end of experiment.
